# Effect of splenectomy on type-1/type-2 cytokine gene expression in a patient with adult idiopathic thrombocytopenic purpura (ITP)

**DOI:** 10.1186/1471-2326-4-4

**Published:** 2004-10-18

**Authors:** Fotios P Panitsas, Athanasia Mouzaki

**Affiliations:** 1 Hematology & Transfusion Medicine, Medical School, University of Patras, Patras, Greece

## Abstract

**Background:**

In view of clinical observations and laboratory results that support a central role of the spleen in idiopathic thrombocytopenic purpura (ITP) pathophysiology, we studied the effect of splenectomy on type-1 and type-2 cytokine gene expression in an adult ITP case, refractory to conservative treatment.

**Case presentation:**

The patient was subjected to splenectomy 9 months after the diagnosis with complete response, attaining platelet counts over 150 × 10^6^/L within 10 days after the operation. Two consecutive blood samples were obtained from the patient, 3 and 7 months after the splenectomy for the purposes of this study. A control group consisted of 11 healthy adults. Peripheral blood mononuclear cells were prepared from each blood sample and cultured *in vitro *for 8 h with the addition of the mitogens phorbol myristate acetate and ionomycin. Total cellular RNA extracted from 10^6 ^cells was submitted to semiquantitave reverse transcriptase-polymerase chain reaction (RT-PCR) for the amplification of IL-2, IFN-γ, IL-4, IL-5, and IL-10 metagraphs. The PCR products were run on ethidium-stained agarose gels, photographed and quantified by densitometry.

A steep decrease of type-1 cytokine expression (IL-2, IFN-γ) and their calculated sum expressing Th1 activity was observed at 7 months post-splenectomy compared to 3 months post-splenectomy, in parallel with a rise of platelet count from 190 × 10^6^/L to 265 × 10^6^/L. The change of type-2 cytokine expression (IL-4, IL-5, IL-10) was slight and the Th2 activity (IL-4+IL-5) remained largely unchanged. The Th1/Th2 ratio, that reflects the pathogenic disease-specific T-cell immune deviation, was accordingly reduced 7 months post-splenectomy (Th1/Th2 = 1.3) compared to 3 months (Th1/Th2 = 3.5).

**Conclusions:**

The reduction of the Th1/Th2 cytokine ratio that was observed over time after splenectomy was accompanied by full clinical remission. Nevertheless, the persistence of a type-1 polarization, even after several months following spleen removal, is suggestive of a more basic abnormality of the immune function in these patients.

## Background

Adult autoimmune thrombocytopenic purpura (ITP) is a chronic acquired organ-specific autoimmune thrombocytopenic syndrome [1]. The low peripheral platelet concentration observed in ITP is the result of reduced platelet life span because of their early removal from the peripheral blood by the activated reticuloendothelial system of the spleen, liver or bone marrow, after their sensitization by autoantibodies that recognize their surface glycoprotein antigens [2]. Apart from phagocytosis, destruction mechanisms include complement activation [3] and direct cellular attack by T lymphocytes [4]. Ineffective thrombocytopoiesis because of autoimmune attack of megakaryocytes in the bone marrow contributes to the thrombocytopenia with varying degrees among cases [2]. The production of platelet autoantibodies by B-cells is driven by activated platelet-specific autoreactive T-cells [5]. The phenotype of the disease-specific T helper cells has been shown to be skewed towards type 1 cytokine production [6-8].

The spleen is considered to be the primary site of the autoimmune response where initiation, maintenance and regulation of the autoimmune attack take place. The spleen is the site of autoreactive T- and B-cell interaction and activation, and autoreactive anti-platelet antibody production [9]. Platelet destruction is also sited mainly in the spleen in most patients [2,10,11].

Splenectomy is followed by reduction of autoantibody peripheral blood titre [12,13]. Spleen cells isolated from ITP patients produce antiplatelet immunoglobulin in *in vitro *cultures [14]. The percentage of T- and B-cells with activated phenotype is far greater in the spleen than in the peripheral blood of ITP patients [9]. The number of circulating autoreactive anti-platelet T- and B-cells declines after splenectomy that leads to clinical remission, whereas the peripheral blood concentration of CD3^+^CD4^+^, CD3^+^CD8^+^, CD3^+^HLADR^+^, and CD3^+^CD25^+ ^cells increases significantly in ITP patients refractory to splenectomy [15]. Changes in the histology of the spleen have been observed in ITP patients and include follicular hyperplasia, foam macrophages, and extramedullary hematopoiesis, among others [16].

Splenectomy is the most clinically effective therapeutic intervention in ITP patients, resulting in complete remission in two thirds of the patients with more than 60% maintaining the therapeutic effect in the long term [17,18]. Irrespective of clinical response, splenectomy seems to affect the natural history of the disease and to enhance the response of ITP patients to other treatments that follow [19].

Given the important immunoregulatory role of the spleen, we looked at the effects of splenectomy on immune activation and immune deviation indices in the peripheral blood of an ITP patient after splenectomy in association with peripheral platelet counts.

## Case presentation

A 42 year old woman presented in October 1999 in Patras University Hospital (PUH) with lower limb purpura and low platelet count (7 × 10^6^/L). Following clinical exclusion of causes of secondary thrombocytopenia [20] the diagnosis of ITP was reached.

The patient initially received glucocorticoid treatment to which she showed a temporary response until 6 months later when she relapsed. She was subsequently started on danazol without any clinical benefit. Intravenous immune globulin administration also proved ineffective after two 5-day cycles. As a result, the patient was subjected to splenectomy 9 months after the diagnosis with complete response, attaining platelet counts over 150 × 10^6^/L within 10 days after the operation. Five years later, she remains in clinical remission.

Two consecutive blood samples were obtained from the patient, 3 and 7 months after splenectomy for the purposes of this study. A control group consisted of 11 adult healthy volunteers (6 women and 5 men, median age 40 years, range 18–65 years). Informed consent was obtained from the patient. PUH abides by the Helsinki declaration on ethical principles for medical research involving human subjects.

Peripheral blood mononuclear cells (PBMC) were prepared from each blood sample by centrifugation over a Ficoll-Paque gradient (Pharmacia, Sweden). The cells were cultured *in vitro *for 8 h with the addition of 20 ng/ml phorbol myristate acetate (PMA) and 1 μM ionomycin (Sigma, St-Louis, MI).

Total cellular RNA extracted from 10^6 ^cells was submitted to semiquantitative RT-PCR for the amplification of IL-2, IFN-γ, IL-4, IL-5, and IL-10 metagraphs [8]. Primers and conditions for the RT-PCR are summarized in Table 1. The PCR products were run on ethidium-stained agarose gels, photographed and quantified [8].

**Table 1:** Primers and conditions for the RT-PCR experiments performed in this study.

**Gene**	**Sequence (5'→3')**	**T (°C)**	**Product (bp)**
IL-2	GCAACTCCTGTCTTGCATTG AATGTGAGCATCCTGGTGAG	59	173
IFN-γ	AGCTCTGCATCGTTTTGGGTTC CAAATATTGCAGGCAGGACAACC	64	492
IL-4	CTGTGCTCCGGCAGTTCTAC ACGTACTCTGGTTGGCTTCC	58	176
IL-5	GCTTCTGCATTTGAGTTTGCTAGCT TGGCCGTCAATGTATTTCTTTATTAAG	59	291
IL-10	ACCCAGTCTGAGAACAGCTGC GTTCACATGCGCCTTGATGTCT	61	260
β2m	CCCCCACTGAAAAAGATGAG TCACTCAATCCAAATGCGGC	56	150

**Figure 1:**
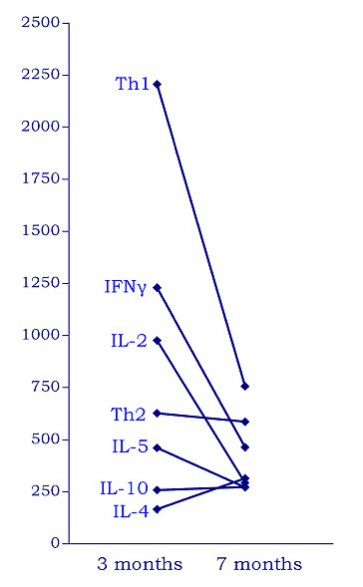
Gene expression levels of individual cytokines and of calculated Th1 and Th2 activities at 3 and 7 months after splenectomy. Th1 equals with IL-2 units plus IFN-γ units and Th2 equals with IL-4 units plus IL-5 units.

**Figure 2:**
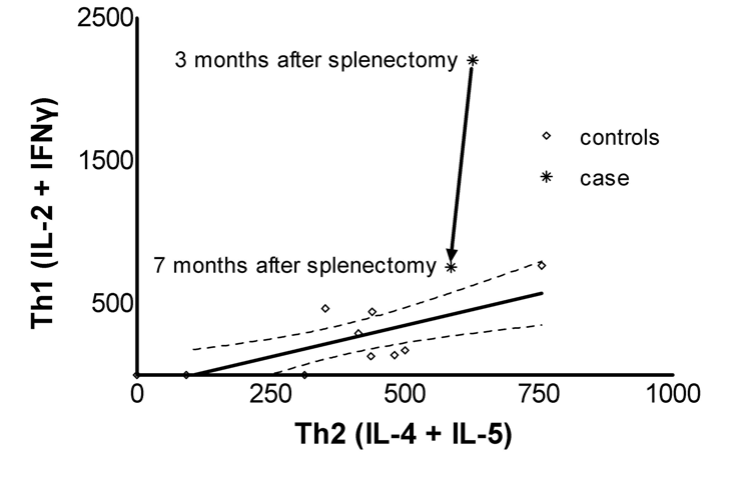
Th1 activity (IL-2+IFNγ) versus Th2 activity (IL-4+IL-5) scatter gram. Asterisks denote the patient's case values at 3 and at 7 months after splenectomy. Diamonds denote the control values. Solid line is the regression line for the controls (r^2 ^= 0.55, p = 0.014) and the two broken lines show the 95% confidence intervals.

A sharp decrease in the expression of the type-1 cytokines IL-2 and IFN-γ and their calculated sum expressing Th1 activity was observed at 7 months after splenectomy compared to 3 months after splenectomy (Figure 1); this was accompanied by a parallel rise of platelet count from 190 × 10^6^/L to 265 × 10^6^/L. Regarding type-2 cytokine gene expression, IL-4 increased, IL-5 decreased, and IL-10 remained unchanged, whereas the change in Th2 activity (IL-4 units plus IL-5 units) was slight (Figure 1). The Th1/Th2 ratio {(IL-2+IFNγ)/(IL-4+IL-5)}, that reflects immune deviation, was accordingly greatly reduced 7 months post-splenectomy (Th1/Th2 = 1.3) compared to 3 months (Th1/Th2 = 3.5) (Figure 2). Mean Th1/Th2 ratio of the controls was 0.5 with 95% confidence intervals of the mean (0.15–0.85). The Th1/Th2 values at 3 months and at 7 months post-splenectomy lie at 6.25 and 1.6 standard deviations above the mean of the controls, respectively.

The above results show that in this patient type-1 polarization persists after removal of the spleen and attainment of clinical remission. This may mean that the spleen is not exclusively responsible for the coordination or the maintenance of the pathological immune response in this patient, provided that no accessory splenic tissue exists. Other disease centres may control the autoimmune reaction as well, such as the liver or the bone marrow. Alternatively, it is possible that ITP is the manifestation of a general immune system malfunction that pre-existed before the development of thrombocytopenia and persists after removal of what seems to be the effector of a manifestation of an autoimmune proclivity.

Unfortunately, a pre-splenectomy sample was not available for analysis. As a result, no conclusions can be drawn about the effect of splenectomy on the direction of change of Th1 activity. Based on phenotypic studies showing increased presence of T lymphocytes with activated phenotype after splenectomy in ITP patients [15], it is plausible that peripheral Th1 activity may have increased after splenectomy.

The clinical remission may be due to the removal of a major platelet destruction site, although the underlying immune activity that drives the destruction may remain unaffected. Complete remission does not mean that increased platelet destruction has stopped after splenectomy. Platelet life span may still be shortened in this patient and/or her normal platelet count may be even higher than what was achieved after splenectomy.

The pathological immune activity seems to decrease over time after splenectomy, as reflected by the lower Th1/Th2 ratio that is indicative of the degree of immune polarization. This may be explained by reduced stimulation of the immune system by activated spleen reticuloendothelial cells that present platelet antigens to T helper lymphocytes. In this way, it may be hypothesized that removal of one vital component of the self-attacking immune process can break the vicious circle that culminates in even greater immune activation, polarization, and platelet destruction. Removal of a major site of autoimmune activity may have abrogated recruitment of naïve T-cells. As a result, overall autoimmune activity wears off, as existing activated Th1 effector cells perish leaving behind a much smaller population of peripheral memory cells that retain the initial Th phenotype.

Another consideration that stems from the results of this case study is that immune polarization and immune deviation of the pathological response depend more on upregulation of type-1 mediators rather than on suppression of type-2 cytokines, or that type-2 response is inadequate to control excess type-1 response in active disease.

## Conclusions

Clinical improvement after splenectomy is associated with reduced but not normalized immune activation and polarization in the patient studied. However, the spleen seems not to be absolutely necessary for the maintenance of the autoimmune reactivity.

## Competing interests

The authors declare that they have no competing interests.

## Authors' contributions

FPP prepared the case report, performed the experiments and drafted the manuscript. AM conceived of the study, participated in its design and coordination and co-wrote the manuscript. Both authors read and approved the final manuscript.

## Pre-publication history

The pre-publication history for this paper can be accessed here:


